# Comparison of the Predictive Value of Blood and Urine Biomarkers During Pregnancy for the Risk of Preterm and Term Pre-Eclampsia

**DOI:** 10.3390/medicina62091617

**Published:** 2026-08-22

**Authors:** Aleksandra Nikolić, Mirjana Bogavac, Nebojša Kladar, Dragan Stajić, Katarina Kovačević, Tamara Popović Perić, Anita Krsman

**Affiliations:** 1 Department of Farmacy, Faculty of Medicine, University of Novi Sad, 21000 Novi Sad, Serbia; nebojsa.kladar@mf.uns.ac.rs; 2 Center of Laboratory Diagnostic and Nuclear Medicine, University Clinical Center of Vojvodina, 21000 Novi Sad, Serbia; lina400kk@gmail.com; 3 Department of Obstetrics and Gynecology, Faculty of Medicine, University of Novi Sad, 21000 Novi Sad, Serbia; mirjana.bogavac@mf.uns.ac.rs (M.B.); dragan.stajic@mf.uns.ac.rs (D.S.); anita.krsman@mf.uns.ac.rs (A.K.); 4 Department of Obstetrics and Gynecology, University Clinical Center of Vojvodina, 21000 Novi Sad, Serbia; 5 PHI Hospital “Sveti Vračevi” Bijeljina, 76300 Bijeljina, Bosnia and Herzegovina

**Keywords:** pre-eclampsia, soluble Fms-like tyrosine kinase-1 (sFlt-1), placental growth factor (PlGF), albumin/creatine ratio

## Abstract

*Background and Objectives*: Pre-eclampsia (PE) is an obstetric complication defined by placental insufficiency, including intrauterine growth restriction and placental abruption. Timely and accurate detection and treatment of pre-eclampsia are difficult because its diagnostic criteria are still based on nonspecific signs and symptoms and because the association between common severity criteria, and unfavorable outcomes for mother and fetus remain unclear. Therefore, the aim of this study was to evaluate the potential use of blood and urine PE risk markers—soluble Fms-like tyrosine kinase-1 (sFlt-1), placental growth factor (PlGF), sFlt-1/PlGF ratio, and albumin/creatin ratio—in predicting and managing PE in groups at risk for preterm and term PE (gestational weeks 24–37) and to correlate the levels of blood and urine PE markers with pregnancy outcome indicators. *Materials and Methods*: This study was conducted at the Department of Obstetrics and Gynecology and at the Center of Laboratory Diagnostics and Nuclear Medicine, University Clinical Center of Vojvodina. This study included 166 pregnant women (GW 24–37) divided into two groups: study group P (n = 52), consisting of pregnant women who were identified as a high-risk group for PE according to clinical signs and medical history and who subsequently developed PE; control group C (n = 114), consisting of pregnant women who had risk factors in their medical history but did not eventually develop PE symptoms. The evaluated parameters included two groups: blood and urine PE risk indicators—biochemical markers such as PlGF, sFlt-1, and sFlt-1/PlGF ratio; urine risk indicators such as albumin/creatine ratio; and pregnancy outcome indicators—gestational age of delivery (GW) and neonatal birth weight (NW). *Results*: The results of the analysis support that the PE risk biochemical indicators (PlGF, sFlt-1, sFlt-1/PlGF ratio, and albumin/creatine ratio) show statistically significant differences between the analyzed groups, that they correlate with PE occurrence, and that they have good predictive value. As expected, sFlt-1/PlGF ratio and albumin/creatine ratio, as the most valuable PE risk indicators, show good predictive value. However, our results also demonstrate that pro- and antiangiogenic indicators alone, especially sFlt-1 (high values), may play roles in risk evaluation and prediction of preterm and term PE (GW 24–37). *Conclusions*: In our study, all evaluated blood and urine indicators (PlGF, sFlt-1, sFlt-1/PlGF ratio, and albumin/creatine ratio) show high predictive value for PE in a PE risk group of pregnancies between GW 24 and 37. The results suggest that sFlt-1 alone has equal predictive value to sFlt-1/PlGF ratio and outperformed PlGF in predicting PE onset. The combined use of pro- and antiangiogenic biochemical markers, indices, and urine markers in blood, together with patient history and clinical parameters, shows potential as a more effective means of predicting PE than a single biochemical parameter such as sFlt-1/PlGF ratio.

## 1. Introduction

Pre-eclampsia (PE) is a progressive multisystem disease that occurs in about 3% of all pregnancies, with hypertensive disorders contributing to complications in 5–10% of all pregnancies [1,2]. Hypertensive disorders during pregnancy are key causes of maternal–fetal morbidity and mortality, being responsible for more than 70,000 maternal deaths and 500,000 fetal deaths every year [2]. Fortunately, late-onset PE, which affects pregnant women over 34 weeks of gestation, is more common than early-onset pre-eclampsia.

Timely and accurate recognition and management of PE are often challenging because diagnostic criteria are still based on nonspecific signs and symptoms and because common severity criteria correlate poorly with adverse maternal and fetal outcomes. Given the heterogeneity of symptoms and clinical presentation, multi-marker models have proven to be successful in predicting PE [3].

The prevailing hypothesis suggests that inadequate uteroplacental vascular remodeling induces placental ischemia and hypoxia, resulting in impaired placental perfusion and endothelial dysfunction in PE [4,5]. Placental dysfunction is characterized by an anomalous trophoblastic invasion of the myometrial segment of the spiral arteries, leading to placental ischemia [6,7]. Consequently, this hypoxic environment promotes the discharge of antiangiogenic factors into maternal circulation, such as soluble Fms-like tyrosine kinase-1 (sFlt-1), and reduces the bioavailability of proangiogenic factors, such as placental growth factor (PlGF). sFlt-1/PlGF ratio as an indicator of placental dysfunction has been widely studied within the last decade [7]. Abnormal levels of sFlt-1 and PlGF have been found to be detected in maternal blood secondary to placental release in cases of placental dysfunction [7,8]. Variations in these angiogenic markers can be detected before clinical manifestations appear. Some studies report an increase in maternal sFlt-1 levels 5 weeks before symptom onset [8,9]. In women with PE, their sFlt-1/PlGF ratio increases rapidly one to four weeks prior to diagnosis, making these markers useful for not only diagnosing but also excluding PE. Biomarker evaluation has been suggested to improve the short-term prediction of preterm PE and could help in deciding when to deliver in the case of term PE [10].

Currently, an sFlt-1/PlGF ratio cut-off level of ≤38 is widely accepted for ruling out suspected PE and a ratio > 85 is accepted as at risk for early and median onset of PE (before GW 34), while a value > 110 is accepted as at risk for late PE (after GW 34) [11]. However, evidence regarding the management and prognosis of women with an abnormally high sFlt-1/PlGF ratio is more limited [12].

Clinical hallmarks currently used to diagnose PE include new onset of hypertension (systolic blood pressure ≥ 140 mmHg or diastolic blood pressure ≥ 90 mmHg) with proteinuria and/or end-organ dysfunction (renal insufficiency, thrombocytopenia, impaired liver function, pulmonary edema, new-onset headache, and/or visual disturbances) after 20 weeks of gestation [13].

Multi-marker modeling using biochemical markers has proven to be more effective in predicting pre-eclampsia and related hypertensive disorders than evaluation of a single parameter, or blood pressure and proteinuria [3,13].

The aims of this study are as follows:

- To evaluate the usefulness of blood and urine PE risk markers (sFlt-1, PlGF, sFlt-1/PlGF ratio, and albumin/creatine ratio) in predicting and managing PE in a high-risk group of patients between 24 and 37 weeks of gestation with present PE risk factors and suspected PE.
- To correlate the levels of available blood and urine risk markers for PE (sFlt-1, PlGF, sFlt-1/PlGF ratio, and albumin/creatin ratio) with pregnancy outcome indicators (gestational age (GW) and neonatal birth weight (NW)) in a high-risk group of patients between 24 and 37 weeks of gestation with PE risk factors and suspected PE.
- To propose a model of PE risk prediction that includes available pro- and antiangiogenic risk markers for PE in blood and urine (sFlt-1, PlGF, sFlt-1/PlGF ratio, and albumin/creatin ratio).

## 2. Materials and Methods

This prospective study was conducted at the Department of Obstetrics and Gynecology and Center of Laboratory Diagnostics and Nuclear Medicine, University Clinical Center of Vojvodina in Novi Sad, Serbia, from May 2024 to January 2026.

### 2.1. Patients

A total of 166 pregnant women agreed to participate in the study, confirmed by written consent in accordance with the criteria of the Declaration of Helsinki.

The inclusion criteria for this study were singleton pregnancy, patient age older than 18, gestational age at 24–37 weeks of pregnancy, and presence of one or more risk factors for PE in the patient’s medical history. The risk factors were recognized as previous PE, chronic hypertension before pregnancy, gestational hypertension, pre-gestational diabetes, gestational diabetes mellitus, obesity (body mass index (BMI) > 30), chronic kidney disease, and cardiovascular disease. All participants were admitted to the Department of High-Risk Pregnancies for further monitoring and followed during pregnancy and delivery. At hospitalization, all participants had medical histories positive for at least one of following symptoms relevant to PE: hypertension or proteinuria during their current pregnancy. The exclusion criteria for this study were multiple pregnancies, patient age younger than 18, and gestational age of <24 weeks of pregnancy.

The sample size was calculated from previous data at the Department of Obstetrics and Gynecology, University Clinical Center of Vojvodina, during a five-year period with 6700 deliveries per year. The mean number of PE patients (GW 24–37) per year was 72. The number of PE marker tests conducted for high-risk pregnancies (sFlt-1/PlGF) in our laboratory department during the one-year period (May 2023/May 2024) was 153. The planned sample size was calculated by considering the known number of PE tests conducted, as well as the known number of PE cases during a 12-month period. We therefore planned for >160 participants and 60–70 PE cases during a 18-month period to account for any exclusion criteria.

Blood pressure was evaluated using our hospital’s standardized protocol. Hypertension was defined as elevated blood pressure (systolic ≥ 140 mmHg or diastolic ≥ 90 mmHg) and classified as chronic hypertension—present before conception/pregnancy and before 20 weeks of gestation (self-reported and then verified during a prenatal visit)—and gestational hypertension—present after 20 weeks of gestation [13].

Proteinuria was defined as a quantitative value of >150 mg of protein in urine within 24 h.

This study involved two groups of women with high-risk pregnancies: study group P (n = 52), consisting of pregnant women who were identified as at high risk for PE—patients who had risk factors for PE or suspected PE at gestational weeks 24–37 and subsequently developed PE within a four-week period; control group C (n = 114), consisting of pregnant women at gestational weeks 24–37 who had risk factors in their medical history but did not develop PE during their current pregnancy.

PE was defined as new-onset hypertension (systolic blood pressure (SBP) ≥ 140 mmHg or diastolic blood pressure (DBP) ≥ 90 mmHg on two occasions, with an interval of 6 h or more) and proteinuria (urinary excretion of ≥300 mg of proteins within 24 h) and/or end-organ dysfunction after 20 weeks of gestation (signs of end-organ damage or uteroplacental dysfunction).

Blood and urine samples were collected and analyzed once, during the first 24 h after patients were admitted to the department. The time period of observation defined between blood and urine sampling, and PE symptom development was four weeks (1–28 days). Group P consisted of patients who developed PE symptoms within the first 24 h after blood and urine sampling, as well as patients who were at risk and then developed PE symptoms later, between days 2 and 28.

### 2.2. Methods

The measured parameters were divided into two groups: PE risk indicators—biochemical markers such as levels of PlGF and sFlt-1 in the blood, and sFlt-1/PlGF ratio—or urine risk indicators such as albumin/creatin ratio; pregnancy outcome indicators—gestational age (GW) and neonatal birth weight (NW).

Biochemical markers of PE (i.e., PlGF, sFlt-1, and sFlt-1/PlGF ratio) were determined in patients’ sera using the ECLIA method with Roche Elecsys Diagnostics immunoassay testing on an Cobas e411 analyzer (Roche Diagnostics), according to the manufacturer’s protocol. Blood samples were collected from the antecubital vein into serum tubes during a 24 h period, centrifuged at 3000× *g*/min, and analyzed on the same day as part of routine clinical care (within 4 h). Risk of PE was determined following recommended criteria (cut-off values of 38 and 85 are accepted for ruling out and ruling in pre-eclampsia, respectively, when using Roche Elecsys assays). The validated lower cut-off value for sFlt-1/PlGF ratio in the short-term prediction of PE is ≤38—rules out PE within one week (NPV of 99%) [14]. The higher cut-off values are >85 (20 to 33 + 6 weeks of gestation/early PE) and >110 (34 + 0 weeks of gestation to delivery/late PE) for sFlt-1/PlGF ratio when testing for PE [11].

Urine samples were collected first thing in the morning, stored at 4–8 °C, and analyzed on the same day as part of routine clinical care (within 2 h). The albumin/creatine ratio was determined from first-morning urine using an automatic system, Alinity C (Abbot Laboratories, Chicago, IL, USA), according to the manufacturer’s protocol and following recommended criteria (reference range cut-off value of ≤3.5 mg/mmol).

Data on gestational age at PE onset, gestational age of delivery (GW), neonatal birth weight (NW), parity, conception method (such as In Vitro Fertilization (IVF)), and mode of delivery (Cesarean section vs. vaginal delivery) were obtained from the patient’s medical history.

### 2.3. Statistics

The results collected in this study were statistically analyzed using Tibco Statistica v13.5 and MedCalc software (version 22.016). This included the application of descriptive and univariate statistical techniques. The normality of the distribution of continuous variables was tested with the Shapiro–Wilk test. Differences between groups were evaluated using the Mann–Whitney U and Chi square tests, depending on the type of studied variable, while Spearman’s rank order and point-biserial correlation were applied to study the relations between variables. Receiver operating characteristic (ROC) analysis was used to evaluate the predictive value of biochemical parameters regarding clinical outcome. The level of statistical significance was set at *p* < 0.05 for all analyses.

## 3. Results

A total of 166 women admitted to the High-Risk Pregnancy Department of Gynecology and Obstetrics were included in our study and defined as the high-risk group (gestational weeks 24–37) based on risk factors for PE or suspected PE. Among them, 52 developed PE (group P), while 114 (control group) did not develop PE symptoms, although they continued to be observed as patients at risk for early or term PE. In group P, the observation time interval was four weeks between blood and urine analyses, and PE symptom development. Although the observation period lasted four weeks, the mean interval (in days) between blood sampling and PE symptom presentation/delivery was remarkably shorter (6.4 days), with 38% of patients in group P developing PE symptoms or delivering within 24 h from blood sampling. In our study, no patients developed PE beyond the four-week period following blood and urine sampling (group P).

The values of the PE risk indicators (PlGF, sFlt-1, sFlt-1/PlGF ratio, and albumin/creatine ratio) and pregnancy outcome indicators (GW and NW) in the PE (P) and control (C) groups are shown in Table 1 (valid N, mean value, median value, minimum value, maximum value, lower and upper quartile, and Std deviation). The difference in PE risk indicators (PlGF, sFlt-1, sFlt-1/PlGF ratio, and albumin/creatine ratio) and pregnancy outcome indicators (GW and NW) in the PE (P) and control (C) groups evaluated based on the Mann–Whitney U test is shown in Table 1.

The analysis of PE risk indicators and pregnancy outcome indicators between groups P and C indicates statistically significant differences for all analyzed parameters: sFlt-1, PlGF, sFlt-1/PlGF ratio, albumin/creatine ratio, GW, and NW (*p* < 0.05). Significantly higher sFlt-1/PlGF ratios, sFlt-1 levels, and albumin/creatine ratios were detected in the PE group (P) relative to the control group (C), while significantly lower PlGF levels were detected in the PE group (P) relative to the control group (C).

Statistically significant differences in GW and NW were also detected between groups P and C, with younger gestational age at delivery and lower NW observed in the PE group (P) compared with the control group (C) (Table 1).

Qualitative parameters relevant for pregnancy outcome were recorded in groups P and C and were analyzed using the Chi-square test (*p* < 0.05): method of conception (IVF conception), presence of hypertension based on medical data at the time of hospitalization, presence of proteinuria based on medical history, and delivery method (Cesarean section vs. vaginal delivery).

Statistically significant differences were detected between groups P and C regarding hypertension incidence (χ2 (1) = 9.786 *p* = 0.0017) and proteinuria incidence (χ2 (1) = 39.26 *p* = 0.00). More incidences of hypertension and proteinuria (at blood sampling) were detected in the medical histories of the PE group (P) than in the control group (C). Statistically significant differences were detected between groups regarding pregnancy outcome incidence, with higher incidence of Cesarean section detected in the PE group (P) (χ2 (1) = 14.37 *p* = 0.0007). No statistically significant differences were detected between groups regarding IVF as a conception method (χ2 (1) = 0.17 *p* = 0.67).

The incidence of IVF, hypertension, proteinuria, each delivery method, and parity in groups P and C are shown in Table 2. In particular, 100% (52 of 52) of subjects in group P and 76.3% (87 of 114) in group C had hypertension in their medical history at the time of hospitalization and blood sampling, while 75% (39 of 52) of subjects in group P and 23.68% (27 of 114) in group C had proteinuria.

Regarding the conception method, IVF was more common in group P than in group C (21.15% vs. 18.42%), and regarding the delivery method, Cesarean section was more common in group P than C (94.23% vs. 65.78%). Nulliparous pregnancy had the same incidence in groups P and C (53.84% vs. 53.50%).

**Figure 1:**
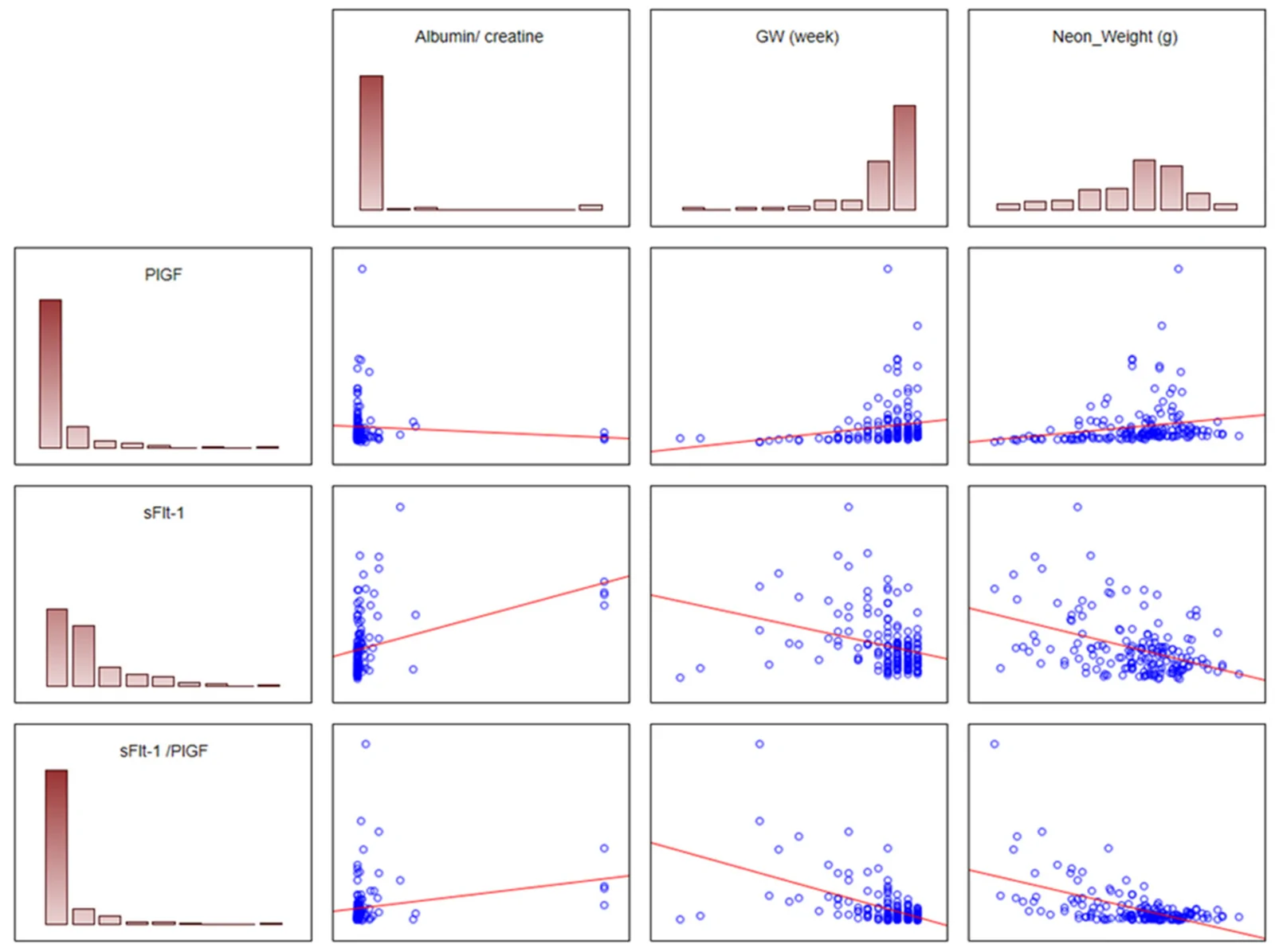
The results from the correlation analysis (plots) between PE risk indicators (PlGF, sFlt-1, sFlt-1/PlGF, and albumin/creatine) and pregnancy outcome indicators (gestational age (GW) and neonatal birth weight (NW)) in the PE (P) and control (C) groups.

Correlation analyses between parameters (PE risk indicators and pregnancy outcome indicators) are shown in Table 3 and Figure 1.

Statistically significant correlations (*p* < 0.05) were detected between all evaluated parameters, except for the correlation between PlGF level and albumin/creatin ratio, which was not statistically significant.

A statistically significant positive correlation was detected between sFlt-1/PlGF ratio and albumin/creatine ratio (rs = 0.41, n = 127, *p* = 0.00). In contrast, a statistically significant negative correlation was detected between sFlt-1/PlGF ratio and the pregnancy outcome parameters: GW (rs = −0.399, n = 164, *p* = 0.00) and NW (rs = −0.463, n = 161, *p* = 0.00).

The highest statistically significant positive correlation was detected between the antiangiogenic marker sFlt-1 and albumin/creatine ratio (rs = 0.542, n = 127, *p* = 0.00), while the correlation between sFlt-1, and the pregnancy outcome parameters GW and NW was statistically significant but negative (rs = −0.364, n = 164, *p* = 0.00) . A statistically significant positive correlation was also detected between the proangiogenic marker PlGF and the pregnancy outcome parameters: GW (rs = 0.340, n = 164, *p* = 0.00) and NW (rs = 0.436, n = 161, *p* = 0.00).

Statistically significant correlations were found between all evaluated biochemical PE indicators and PE occurrence: Statistically significant correlations were detected between the PE indicator sFlt-1/PlGF ratio and PE occurrence (rpb (166) = 0.40, *p* < 0.00) and between the PE indicator sFlt-1 and PE occurrence (rpb (166) = 0.50, *p* < 0.00), while the correlation between PlGF-1 and PE occurrence was statistically significant but negative (rpb (166) = −0.20, *p* = 0.006). A statistically significant correlation was also detected between the PE risk indicator albumin/creatine ratio and PE occurrence (rpb (127) = 0.28, *p* = 0.001). Comparing the correlation index values, the highest correlation values detected were between sFlt-1 and PE occurrence (0.50).

**Figure 2:**
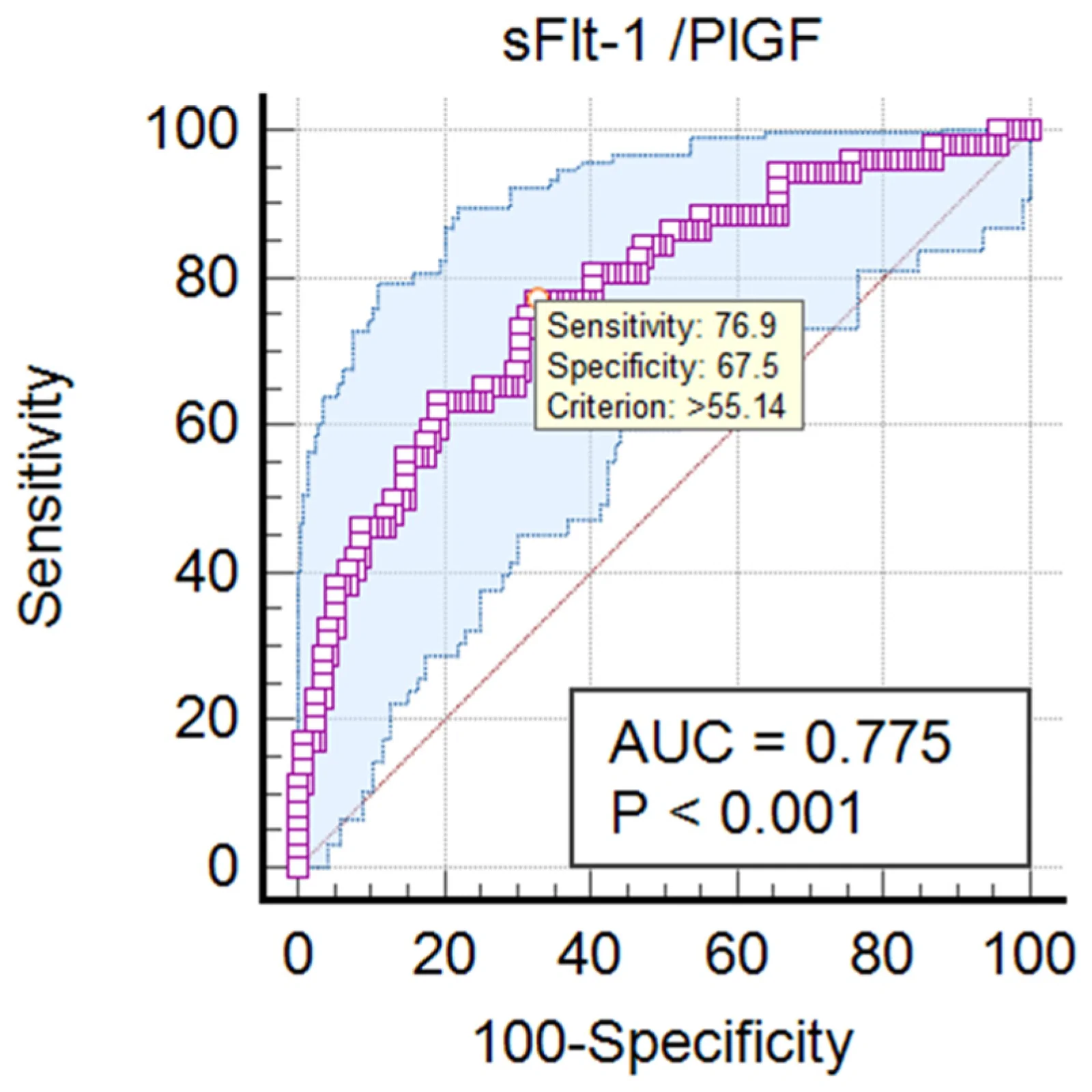
The result of the ROC analysis of sFlt-1/PlGF ratio and prediction of PE.

**Figure 3:**
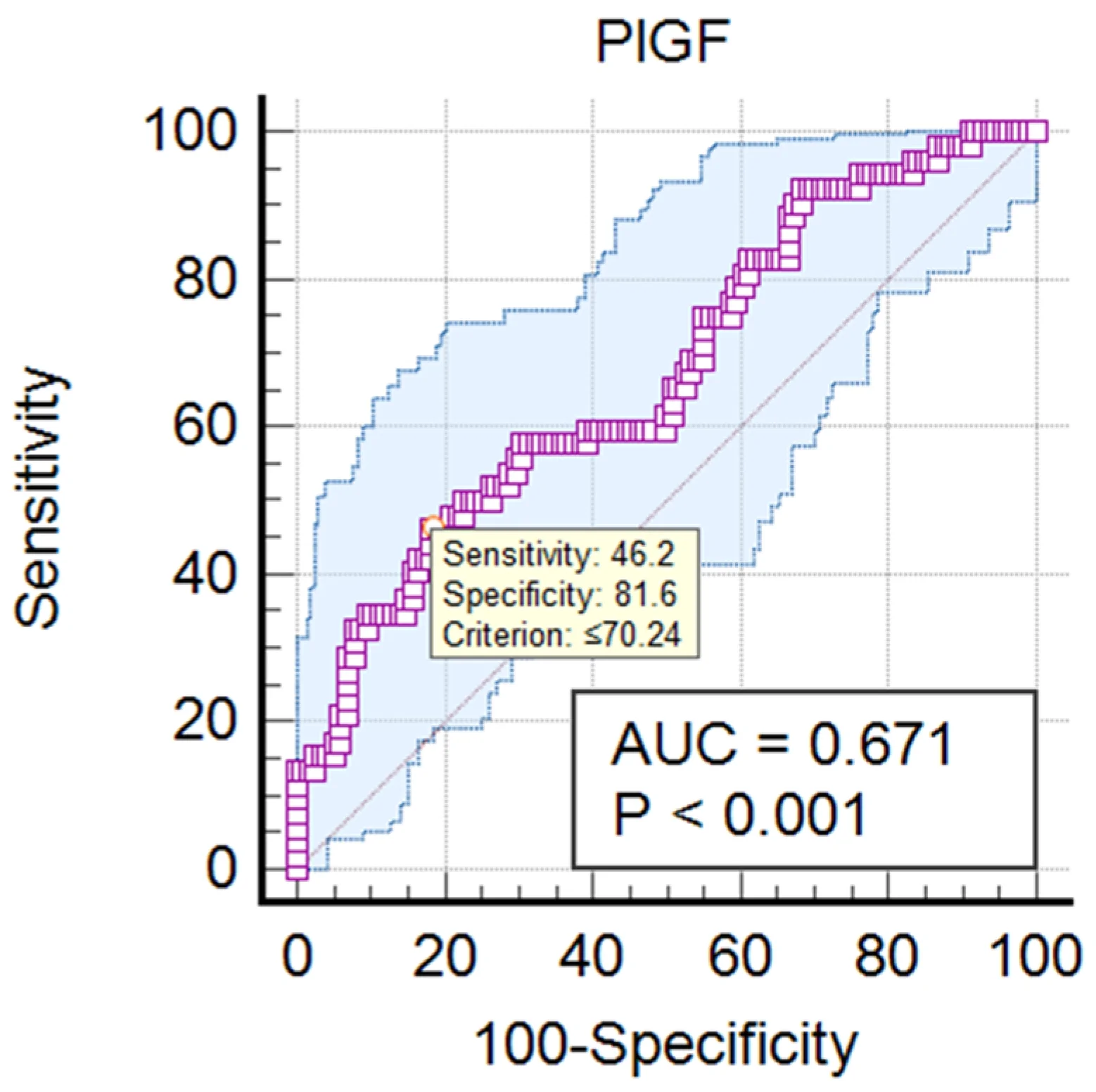
The result of the ROC analysis of PlGF and prediction of PE.

**Figure 4:**
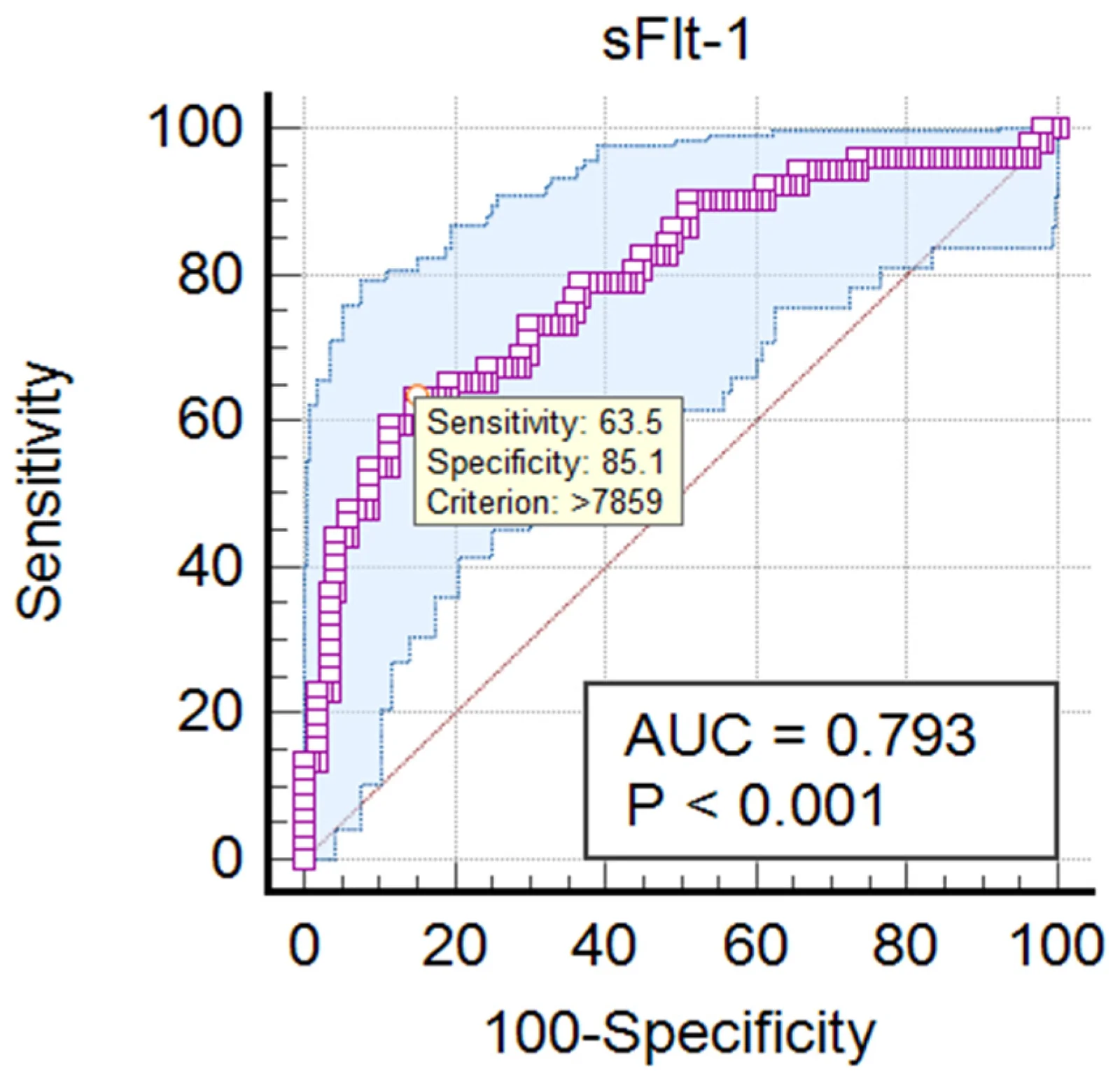
The result of the ROC analysis of sFlt-1 and prediction of PE.

ROC (receiver operating characteristic) curves for the prediction of PE using the sFlt-1/PlGF ratio, and PlGF and sFlt-1 levels are presented in Figure 2, Figure 3 and Figure 4.

According to the ROC analysis, the sFlt-1/PlGF ratio demonstrated good prediction performance regarding PE, with an AUC of 0.775. At the optimal threshold of 55.14, the test achieved 76.9% sensitivity and 67.5% specificity (Figure 2). PlGF level also demonstrated good prediction performance regarding PE, with an AUC of 0.671. At the optimal threshold of PlGF values < 70.24 pg/mL, the test achieved 46.2% sensitivity and 81.6% specificity (Figure 3).

Additionally, sFlt-1 level demonstrated good prediction performance regarding PE, with an AUC of 0.793. At the optimal threshold of 7859 pg/mL, the test achieved 63.5% sensitivity and 85.1% specificity (Figure 4).

**Figure 5:**
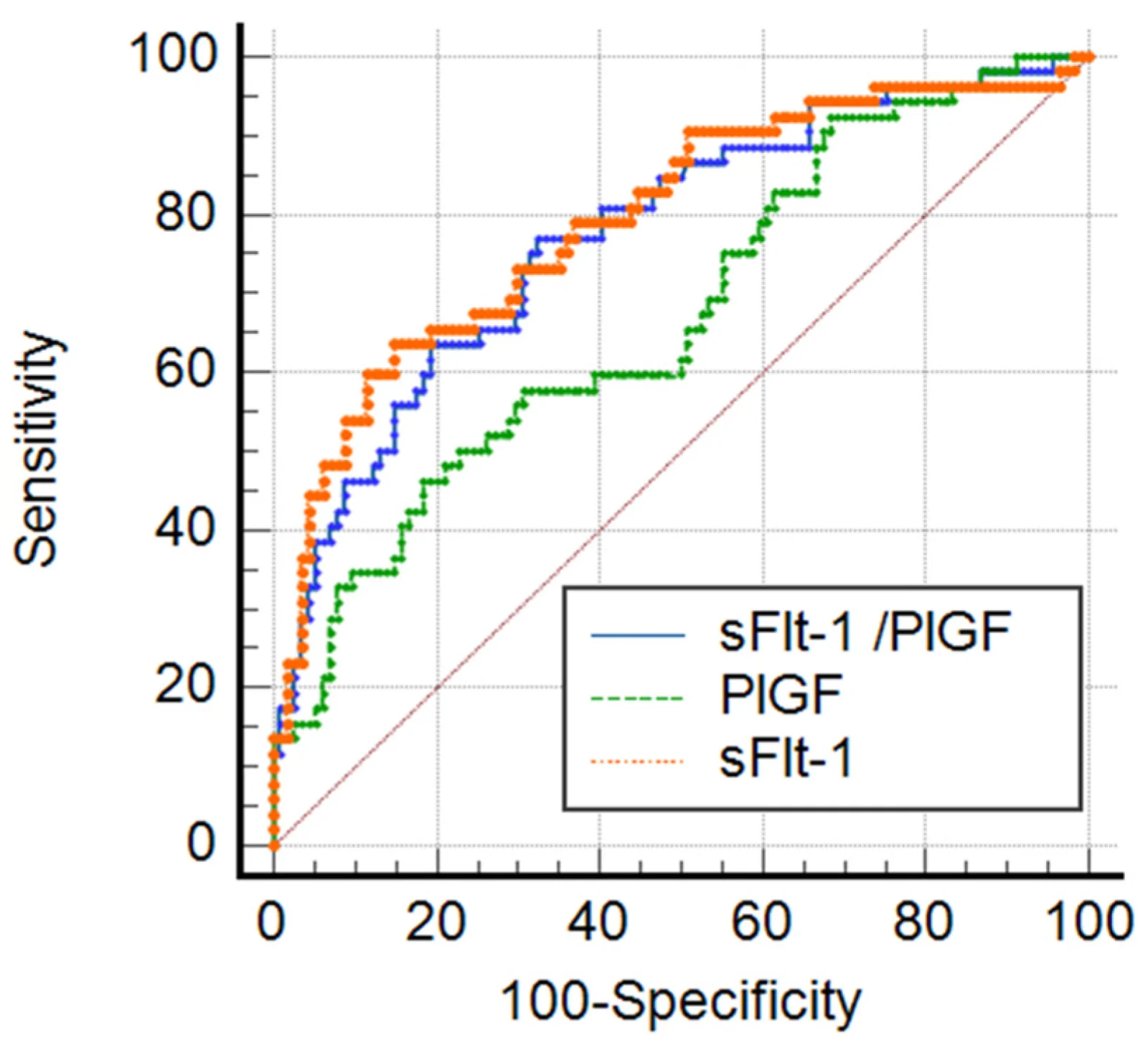
The result of the ROC analysis of sFlt-1/PlGF, sFlt-1, PlGF, and prediction of PE.

No statistically significant difference was detected between the prediction values of sFlt-1 level and sFlt-1/PlGF ratio (z = 0.701, *p* = 0.48) in relation to PE (Figure 5). In contrast, statistically significant differences in prediction value were found between sFlt-1 and PlGF levels (z = 2.58, *p* = 0.01), as well as between the sFlt-1/PlGF ratio and PlGF level (z = 3.94, *p* = 0.00).

## 4. Discussion

We evaluated the use of pro- and antiangiogenic biomarkers, as well as urine biomarkers in high-risk pregnancies at 24–37 weeks of gestation for the early identification of pregnancies with PE outcome. Pregnant women in our study had suspected PE or at least one risk factor for PE present in their medical history and at least one of the symptoms (hypertension and proteinuria), meeting the criteria for acceptance at the High-Risk Pregnancy Department at University Clinical Center of Vojvodina.

In our study, all evaluated blood and urine indicators (PlGF, sFlt-1, sFlt-1/PlGF ratio, and albumin/creatine ratio) showed high predictive values for PE as single markers in a group of patients with pregnancies between GW 24 and 37 at risk of PE. The results confirmed that PE risk biochemical indicators (PlGF, sFlt-1, sFlt-1/PlGF ratio, and albumin/creatine ratio) showed statistically significant differences between groups P and C. The levels of PE risk indicators available in blood and urine correlate with pregnancy outcome indicators. The results of this study therefore support our initial hypothesis.

A healthy pregnancy group was not included in our study, as analyses of PE biomarkers in healthy pregnancies during their second and third trimesters (GW 24–37), and studies on the predictive value of biomarkers and their comparisons with high-risk pregnancies have already been the subject of many studies. Reference intervals for such markers in a healthy pregnancy group have also been established and documented for several methods. During clinical use of PE biomarkers and interpretation of the results, however, we experienced problems whereby patients were recognized as high-risk patients for PE but an analysis of their blood PE biomarkers (preferably sFlt-1/PlGF) did not recognize the risks because their PE biomarker levels were higher than or very close to the cut-off values. Despite these limitations, groups P and C consisted of high-risk pregnancies, with the control group defined as patients who did not eventually develop PE. Thus, one of the aims of our study was to compare the levels of blood and urine risk markers in these two groups of at-risk patients and to recognize the potential differences.

The analysis of PE risk biochemical indicators between groups P and C showed statistically significant differences between groups regarding all analyzed PE risk parameters, indices, and single parameters: sFlt-1, PlGF, sFlt-1/PlGF ratio, and albumin/creatine ratio. As expected, higher mean and median values of the sFlt-1/PlGF ratio and sFlt-1 alone were detected in the PE group compared with the control group, while lower mean and median values of PlGF were detected in the PE group than the control group. This finding is consistent with those from most studies analyzing the same group of indicators [7,14,15,16,17]. Although in routine diagnostics protocols, estimated maternal and fetal risks and the sFlt-1/PlGF, and albumin/creatine ratios are the most valuable PE risk markers, together with clinical signs, our study has shown that pro- and antiangiogenic indicators alone—PlGF (low values) and sFlt-1 (high values)—may also play roles in PE risk evaluation during the second and third trimesters (gestational weeks 24–37), as well as in the evaluation between PE and other forms of related hypertensive disorders, as 76.3% of patients in group C had hypertension during actual pregnancy, together with other risk factors. This finding is consistent with that found by Bastiansen et al. [16], who found that PlGF alone might play a role in the evaluation of early PE risk in a population with PE and addition risk factors (GW 24–29).

The large difference in mean values of sFlt-1/PlGF between groups P and C (238.5 vs. 62) in our study still shows a very high mean ratio in group P, a much higher value than the accepted risk values for preterm (>85) and term PE (>110). At the same time, the mean sFlt-1/PlGF ratio in group C lies between 38 and 85 (62). The angiogenic profile in pregnancies complicated by chronic hypertension, gestational diabetes, insulin-dependent diabetes, or other PE risk factors may therefore reflect a combined effect of metabolic dysregulation, placental compensation, and endothelial dysfunction, but in the control group in our study, these mechanisms still managed to reduce the typical antiangiogenic shift characteristic of PE.

Stepan et al. [12] confirmed that the evidence is more limited regarding the management and prognosis of patients with an abnormally high sFlt-1/PlGF ratio. In our study, a correlation was found between high sFlt-1/PlGF ratio values and unfavorable pregnancy outcome in group P.

A statistically significant difference was found between the mean and median values for the pregnancy outcome parameters in the PE and control groups. In patients from group P, pregnancies finished earlier and, as expected, neonates had lower NW. The correlation analysis showed that anti- and proangiogenic factors alone, high sFlt-1 values, and low PlGF values are consistent with earlier pregnancy termination and lower NW in the PE and control groups. Nonetheless, the higher sFlt-1/PlGF and albumin/creatine ratios show valuable correlation with earlier pregnancy termination and lower NW in both groups.

Additionally, by comparing the risk parameters with PE occurrence, the highest correlation was obtained with high values of sFlt-1 alone (0.50) when compared with the respective ratio values in blood and urine (i.e., sFlt-1/PlGF or albumin/creatin ratios). High values of sFlt-1 as a single parameter might therefore play a role in the evaluation of early and term PE risk.

According to the ROC analysis, sFlt-1 alone showed the best performance regarding PE prediction, compared with sFlt-1/PlGF or PlGF, with AUC values of 0.793, 0.775, and 0.671, respectively (Figure 2, Figure 3, Figure 4 and Figure 5). No statistically significant difference was detected between the prediction values of sFlt-1 level and sFlt-1/PlGF ratio regarding PE. In contrast, statistically significant differences in the prediction values were found between sFlt-1 and PlGF levels, as well as between sFlt-1/PlGF ratio and PlGF level. The results of our study suggest that sFlt-1 alone has equal predictive value to sFlt-1/PlGF ratio for predicting PE onset between GW 24 and 37. Similarly, other studies comparing the predictive values of pro- and antiangiogenic markers confirmed that independent markers may have better predictive values than sFlt-1/PlGF ratio. In Bjorkman’s study (GW 24–29), the ROC analysis for predicting preterm PE demonstrated the best performance with PlGF, compared with sFlt-1 or sFlt-1/PlGF ratio, with AUC values 0.76, 0.49, and 0.70, respectively [17]. The predictive value for sFlt-1/PlGF obtained an AUC of 0.775 in our study, slightly better compared with the 0.70 in Bjorkman’s study [18]. Additionally, in a Danish study investigating angiogenic biomarkers in 1909 patients (137 PE) at GW 20–34, PlGF outperformed the sFlt-1/PlGF ratio when predicting late-onset PE, with an AUC of 0.74 vs. 0.69. Used as an independent risk marker, PlGF showed the best predictive value in Bjorkman’s study and the Danish study, but in contrast, our results showed better predictive value for sFlt-1 compared with PlGF [17,18]. However, our study was conducted only on pregnancies at high risk for PE and did not include healthy pregnancies, but the AUC value for sFlt-1/PlGF ratio in our study is relatively close to that detected in studies conducted on PE risk and healthy pregnancies (0.775 vs. 0.70 and 0.69) [17,18].

### Limitations

There are a few limitations to our study: Our study was conducted only on patients with pregnancies at high risk for PE hospitalized at our department and did not include a comparison with a healthy pregnancy group, only with a control group with PE risks. Variables such as anthropometric data and age were also not included in our study.

## 5. Conclusions

In our study, all evaluated blood and urine indicators (PlGF, sFlt-1, sFlt-1/PlGF ratio, and albumin/creatine ratio) showed high predictive value for PE in a PE risk group of pregnancies between GW 24 and 37. All evaluated blood and urine indicators correlated with the pregnancy outcome indicators (GW and NW). Although the sFlt-1/PlGF and albumin/creatine ratios are the most valuable PE risk indicators relative to all other laboratory parameters and both showed good predictive value for PE, our study shows that pro- and antiangiogenic indicators alone, especially sFlt-1 (high values), might play roles in the evaluation of preterm and term PE risk (GW 24–37). Single indicators, especially sFlt-1, might play an independent role in the evaluation between PE and other hypertensive disorders in a population at risk for PE. The results from our study suggest that sFlt-1 as a single parameter has equal predictive value to the sFlt-1/PlGF ratio for predicting PE onset between GW 24 and 37.

Therefore, the combined use of pro- and antiangiogenic biochemical markers, indices, and urine markers in blood, together with patient history and clinical parameters, show potential as a more effective means of predicting PE than a single biochemical parameter such as the sFlt-1/PlGF ratio.

## Figures and Tables

**Table 1:** The values (mean, median, minimum, and maximum values; lower and upper quartile; and standard deviation) of PE risk indicators (PlGF, sFlt-1, sFlt-1/PlGF, and albumin/creatine) and pregnancy outcome indicators (gestational age (GW) and neonatal birth weight (NW)) in the PE (P) and control (C) groups. The Shapiro–Wilk test indicated that the studied variables were not normally distributed. The statistically significant differences (*p* < 0.05) between groups evaluated using the Mann–Whitney U test are marked with *.

Variable	Group	N	Mean	Median	Min.	Max.	Lower Quartile	Upper Quartile	Std. Dev.	Variable Distribution	Difference Between Groups
PlGF * (pg/mL)	C	114	247.0	115.35	30.09	2311	77.37	273.80	334.2	W = 0.60 *p* = 0.00	U = 1950 *p* = 0.00
	P	52	116.2	77.19	11.22	664.9	44.34	146.1	118.1	W = 0.71 *p* = 0.00	
sFlt-1 * (pg/mL)	C	114	5348.1	4302	488.2	19,986	2653	73,810	3651.7	W = 0.84 *p* = 0.00	U = 1228 *p* = 0.00
	P	52	11,815.3	9852	1010	37,503	5892.5	16,218.5	7616.7	W = 0.93 *p* = 0.00	
sFlt-1/PlGF *	C	114	62.8	36.13	1.14	535.49	12.48	74.79	85.9	W = 0.65 *p* = 0.00	U = 1333.50 *p* = 0.00
	P	52	238.5	119.6	2.75	1706.76	56.26	269.26	310.9	W = 0.69 *p* = 0.00	
Albumin/creatine * (mg/mmol)	C	81	16.0	1.50	0.17	452.8	0.900	3.70	55.1	W = 0.29 *p* = 0.00	U = 678 *p* = 0.00
	P	46	226.7	26.1	0.6	2000	4.5	101.90	580	W = 0.42 *p* = 0.00	
GW * (week)	C	114	37.5	38	24	40	37	39	3.53	W = 0.59 *p* = 0.00	U = 1635.50 *p* = 0.00
	P	52	35.1	37	24	40	33	38.00	4.1	W = 0.84 *p* = 0.00	
NW * (g)	C	114	2918.7	3025	720	4550	2600	3420	742.8	W = 0.97 *p* = 0.00	U = 2098 *p* = 0.01
	P	52	2456.3	2680	350	3810	1774	3170	979	W = 0.94 *p* = 0.02	

**Table 2:** The incidence of IVF pregnancy, delivery method (Cesarean section or vaginal delivery), hypertension in pregnancy, proteinuria in pregnancy, and nulliparous pregnancy in the PE (P) and control (C) groups.

		IVF		Cesarean S.		Vaginal D.		Hypertension		Proteinuria		Nulliparous p.	
	Cumulative Count	Count	(%)	Count	(%)	Count	(%)	Count	(%)	Count	(%)	Count	(%)
Group C	114	21	18.42	75	65.78	39	34.21	87	76.3	27	23.68	61	53.50
Group P	52	11	21.15	49	94.23	3	5.76	52	100	39	75	28	53.84

**Table 3:** The results from the correlation analysis between PE risk indicators (PlGF, sFlt-1, sFlt-1/PlGF, and albumin/creatine) and pregnancy outcome indicators (gestational age (GW) and neonatal birth weight (NW)) in the PE (P) and control (C) groups (tests marked with * are significant at *p* < 0.05).

	Valid N	Spearman’s R	T (N-2)	*p*-Value
PlGF and Albumin/Creatine	127	−0.172508	−1.95805	0.052451
PlGF and GW (weeks) *	166	0.340213	4.60489	0.000008 *
PlGF and NW (g) *	164	0.436900	6.12455	0.00000 *
sFlt-1 and Albumin/Creatine *	127	0.542461	7.21943	0.000000 *
sFlt-1 and GW (weeks) *	166	−0.377597	−5.19026	0.000001 *
sFlt-1 and NW (g) *	166	−0.364047	−4.92866	0.000002 *
sFlt-1/PlGF and Albumin/Creatine *	127	0.402970	4.92272	0.000003 *
sFlt-1/PlGF and GW	166	−0.399341	−5.54403	0.000000 *
sFlt-1/PlGF and NW (g) *	166	−0.463743	−6.60020	0.000000 *

## Data Availability

All data are available within the main document.
